# Vascular leak in sepsis: physiological basis and potential therapeutic advances

**DOI:** 10.1186/s13054-024-04875-6

**Published:** 2024-03-23

**Authors:** Ross R. McMullan, Daniel F. McAuley, Cecilia M. O’Kane, Jonathan A. Silversides

**Affiliations:** 1 Wellcome‐Wolfson Institute for Experimental Medicine, Queen’s University of Belfast, Lisburn Road, Belfast, BT9 7BL UK; 2 Department of Critical Care, Belfast Health and Social Care Trust, Belfast, UK

**Keywords:** Sepsis, Endothelial dysfunction, Oedema

## Abstract

Sepsis is a life-threatening condition characterised by endothelial barrier dysfunction and impairment of normal microcirculatory function, resulting in a state of hypoperfusion and tissue oedema. No specific pharmacological therapies are currently used to attenuate microvascular injury. Given the prominent role of endothelial breakdown and microcirculatory dysfunction in sepsis, there is a need for effective strategies to protect the endothelium. In this review we will discuss key mechanisms and putative therapeutic agents relevant to endothelial barrier function.

## Introduction

Sepsis is a state of organ dysfunction caused by a dysregulated host immune response to infection [1]. Despite advances in medical care, sepsis remains a leading cause of death, accounting for more than 20% of global deaths [2]. A hallmark feature of sepsis is microcirculatory dysfunction which manifests as areas of heterogenous or absent blood flow due to dysregulation of vascular tone, shunting of blood directly from arterioles to venules, and microthromboses [3]. Another key feature of sepsis is enhanced endothelial permeability which leads to interstitial oedema [4]. While this initial increased endothelial permeability is likely beneficial to the host immune response by allowing the transvascular flux of antibodies and antibacterial peptides, ultimately this becomes harmful [4, 5].

Endothelial dysfunction is a common feature of acute inflammatory disorders including burns, trauma, and acute respiratory distress syndrome (ARDS) including that caused by COVID-19, as well as sepsis, and may account for overlap in clinical features between these syndromes.

### Endothelial structure and function

The vascular tree is lined by a monolayer of endothelial cells which are critical to vascular integrity, haemostasis, vasomotor control, and immunological defence via exocrine, paracrine, and autocrine actions [6, 7]. The luminal surface is coated with the endothelial glycocalyx, a gel-like matrix of proteoglycans and glycoproteins [8]. In humans, estimates of endothelial surface area vary between 270 and 7000 m^2^ [9, 10].

A key mediator of vascular tone is nitric oxide (NO), which is synthesised in endothelial cells [11]. NO production is modulated by endothelial shear stress and by various signalling molecules, such as bradykinin, adenosine, serotonin, and vascular endothelial growth factor (VEGF) [12, 13]. Due to the pervasive role of dysregulated NO activity in sepsis, many attempts have been made to correct the heterogenous imbalance of NO in sepsis, all of which have failed to demonstrate benefit [14–17].

Endothelial cells also produce prostacyclin which, in addition to contributing to vasodilation, prevents platelet deposition on the vessel wall [18]. The endothelium produces potent vasoconstrictors such as Endothelin-1 [19] and facilitates the conversion of Angiotensin-1 into Angiotensin-2, another potent vasoconstrictor which is a product of the renin–angiotensin–aldosterone system [20].

### Endothelial cell–cell junctions

Complex inter-endothelial junctional structures, such as adherens junctions and tight junctions, perform a critical role in maintaining vascular integrity and allow endothelial cells to communicate with surrounding structures. The organisation of endothelial cell–cell junction complexes varies along the vascular tree [21]—for example, endothelial junctions in the brain are rich in tight junctions which ensure strict control of permeability across the blood brain barrier [22]. This contrasts with poorly organised tight junctions located in postcapillary venules which readily permit extravasation of inflammatory and immune cells [21, 23].

Adherens junctions are responsible for regulation of cell–cell adhesion, the actin cytoskeleton and intra-cellular signalling [24] and are composed of the core transmembrane protein vascular-endothelial (VE)-cadherin which interacts with cytoplasmic proteins known as catenins. In sepsis, the extracellular domain of VE-cadherin is subject to proteolysis by neutrophil elastase [25] and metalloproteinases [26].

VE-cadherin junctions are tightly regulated by Rho proteins, a subfamily of small GTPases which belong to the Ras superfamily [27]. Key subtypes of the Rho subfamily include Rac1 and RhoA which have been identified to perform central roles in the maintenance of endothelial barrier integrity. The carefully balanced activation of Rac1 and inhibition of RhoA stabilises the VE-cadherin complex and prevents vascular leakage [28]. In experimental models of sepsis, this balance is lost, and impairment of Rho-associated pathways has been identified in endothelial cells [27]. Rac1 activation and RhoA inhibition are associated with VE-cadherin stabilisation and reduced vascular leakage in lipopolysaccharide (LPS) and interleukin (IL)-1*β* models of endothelial dysfunction [29, 30].

**Fig. 1:**
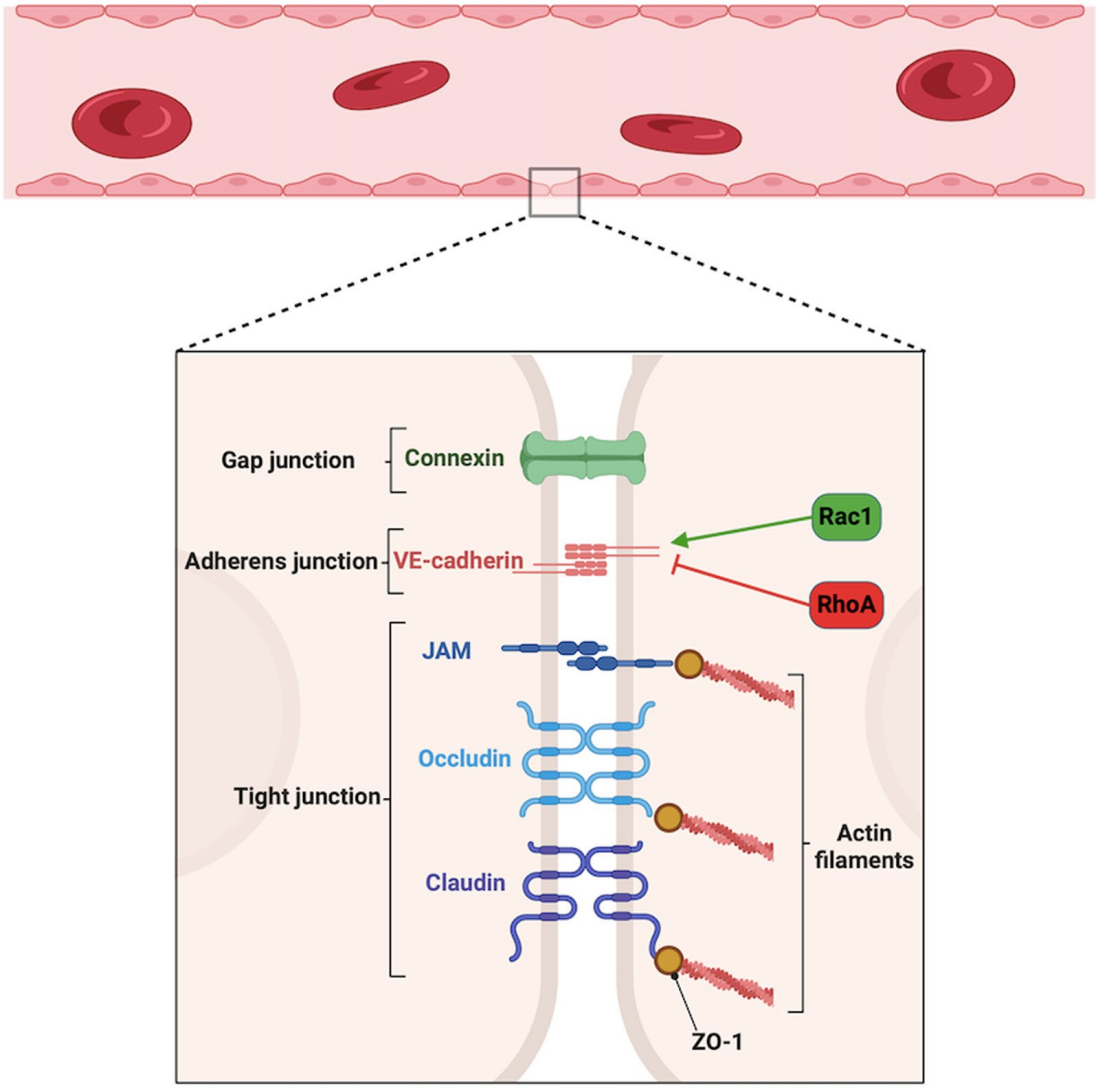
Endothelial cell–cell junction complexes. These key junctional structures maintain endothelial barrier integrity. The ZO proteins link the membrane proteins to the filamentous cytoskeleton. Members of the Rho family of GTPases mediate opposing changes in endothelial cell permeability with Rac1 stabilising the VE-cadherin complex and RhoA de-stabilising the VE-cadherin complex

Tight junctions serve to form a continuous intercellular barrier between cells and act to control the paracellular movement of ions and solutes [24, 31]. Tight junctions are composed of adhesion molecules, such as claudin, occludin and junction adhesion molecules, which exist in complex with the cytoplasmic scaffolding proteins zonula occludens (ZO)-1,-2 and -3 (Fig. 1) [24, 32]. The ZO scaffolding proteins link tight junctions to the actin cytoskeleton either through a direct link or through further protein interactions [24]. ZO-1 has multiple domains which permit a wide array of cellular signalling, thereby providing plasticity of tight junction function [33, 34].

In addition to the key role of adherens junctions and tight junctions in maintaining vascular homeostasis, connexins perform a vital role in intercellular communication. Connexins are transmembrane proteins which form intercellular channels and connect the cytoplasms of adjacent cells, thereby allowing the exchange of ions and small metabolites [35].

**Fig. 2:**
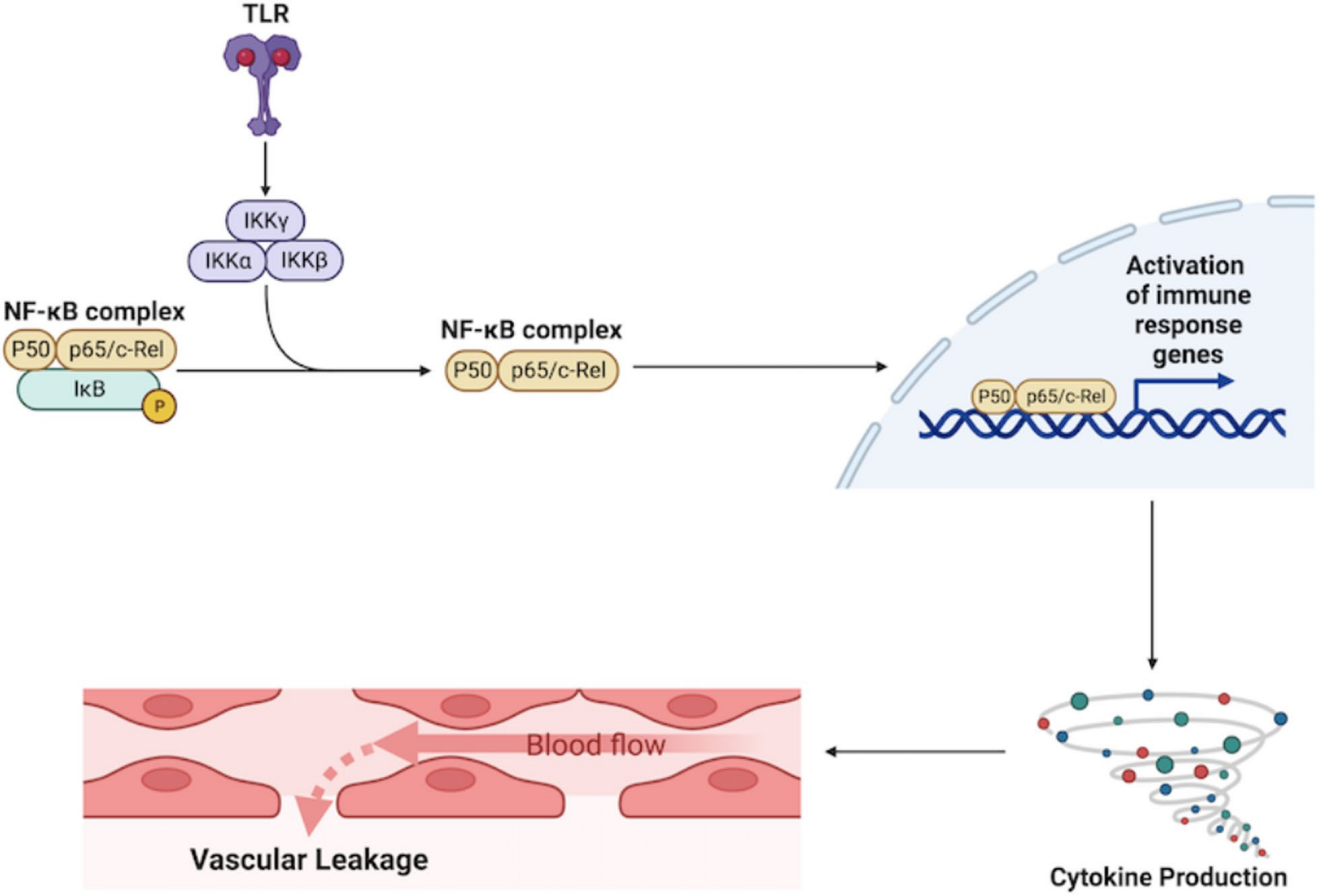
An array of microbial components stimulate the innate immune response by activating Toll-like receptors which results in the nuclear translocation of the transcription factor NF-κB. NF-κB then promotes the expression of pro-inflammatory cytokines such as TNF- *α* which induces endothelial cell dysfunction

Disruption of key adhesion molecules is mediated by TNF-*α* and IL-1*β*, key pro-inflammatory cytokines in sepsis, whose production is increased as a result of activation of NF-κB dependent transcription [36]. In septic patients, NF-κB activity correlates with the severity of illness and is significantly higher in non-survivors [37]. NF-κB activation performs a crucial role in the pathophysiology of sepsis by mediating the inflammatory response via the production of key cytokines, such as TNF-*α* (Fig. 2) [38].

In experimental models of sepsis, the NF-κB pathway is stimulated with the use of LPS, a component of the outer membrane of Gram-negative bacteria [39]. LPS performs a key role in driving Gram negative sepsis [40, 41] by activating Toll-like receptor (TLR) signalling. Ultimately, this cascade enables the nuclear translocation of key transcription factors, such as NF-kB in order to promote pro-inflammatory cytokine gene transcription [42, 43].

TNF-*α* is perhaps the most extensively studied pro-inflammatory cytokine. Tracey and colleagues confirmed that the administration of recombinant TNF-*α* can induce shock and tissue injury [44]. Moreover, it has been demonstrated that the administration of anti-TNF antibodies could prevent shock, organ dysfunction and death in a baboon Escherichia coli model of sepsis [45]. However, despite promising pre-clinical evidence the use of anti-TNF-*α* therapies has proven disappointing in clinical trials [46, 47].

 VEGF is a potent angiogenesis factor and pro-permeability mediator which is produced by endothelial cells and macrophages among a variety of cell types [48]. VEGF expression is primarily promoted by hypoxia [49], but also by pro-inflammatory cytokines such as IL-1 [50], IL-1*β* and TNF-*α* [51]. VEGF is thought to promote endothelial cell permeability via a range of mechanisms. Firstly, it has been demonstrated that the treatment of endothelial cells with VEGF results in the development of, previously absent, fenestrations [52, 53]. Secondly, VEGF results in the formation of clusters of vesicles which link the luminal and abluminal surfaces of endothelial cells. These clusters have been termed vesicular vacuolar organelles and are thought to form a pathway for the transcellular movement of fluid and solute [54, 55]. Finally, VEGF may directly interfere with key endothelial junctional structures. Using immunofluorescence based techniques Kevil and colleagues revealed that endothelial cell treatment with VEGF resulted in a loss of VE-cadherin and occludin [56].

### Endothelial glycocalyx

The glycocalyx, a mesh-like network of proteoglycans and glycoproteins, lines the vascular endothelium [57], and regulates capillary and interstitial oncotic pressures to modulate fluid filtration [58, 59]. Restriction of the transvascular movement of large, negatively-charged molecules such as albumin results in an albumin gradient which opposes fluid flux across the endothelium [60].

**Fig. 3:**
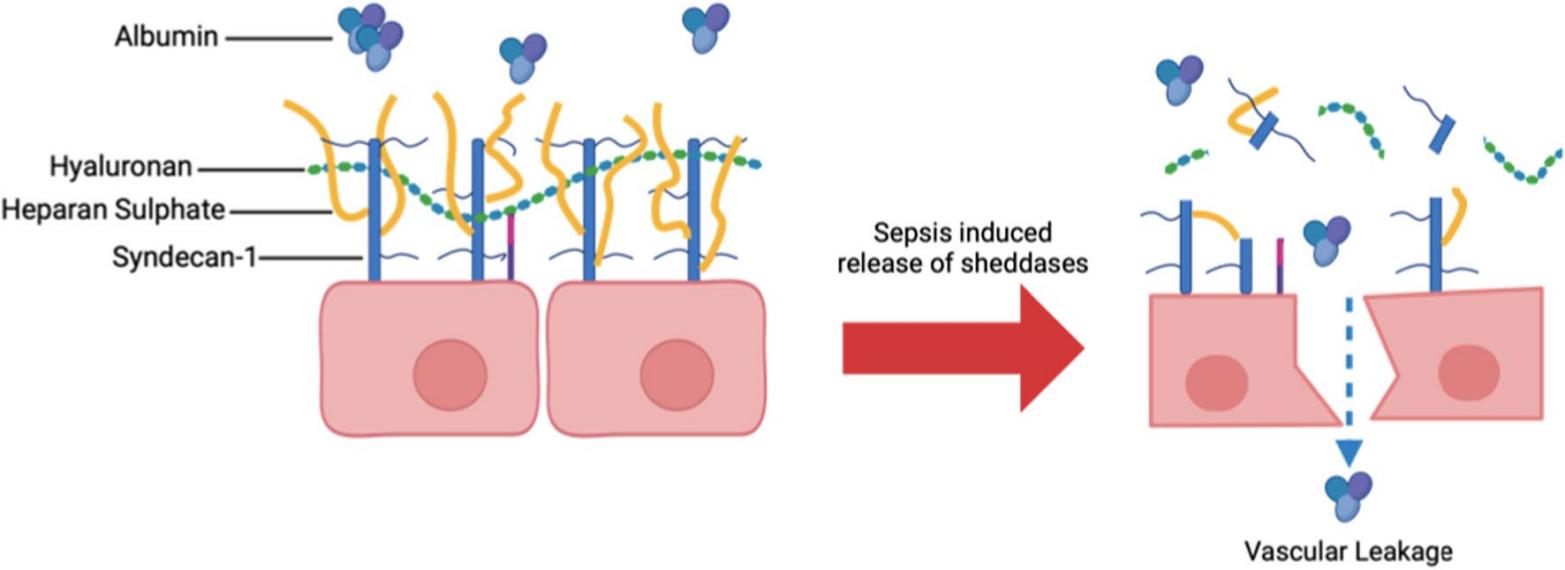
The sepsis state results in vascular leakage due to a combination of glycocalyx degradation and cell–cell disruption. The loss of glycocalyx and endothelial integrity results in the transvascular loss of albumin which favours vascular leakage

In sepsis, degeneration of the glycocalyx results in vascular leak, impaired perfusion, aberrant coagulation and leucocyte activation and adhesion [61–63]. This glycocalyceal degeneration is mediated by sheddases, enzymes such as heparinase and metalloproteinases, which are activated by inflammatory cytokines, such as TNF-*α*, and by Reactive Oxygen Species (ROS) [64, 65], and which cleave the key glycocalyx components heparan sulphate and syndecan-1, respectively [64, 66]. Cleavage of these important glycocalyx components and breakdown of intercellular junctions contributes to vascular leakage (Fig. 3). Since glycocalyceal function includes prevention of platelet adhesion and leucocyte activation and adhesion, injury to the glycocalyx can cause a self-perpetuating cycle of inflammation and further endothelial injury.

Several studies have demonstrated that soluble markers of glycocalyx breakdown, such as syndecan-1, hyaluronan and heparan sulphate, are associated with sepsis presence, severity, and mortality [67–69].

Intravenous fluid therapy, a key component of sepsis resuscitation, may exacerbate glycocalyceal injury [70–72]. The hormone atrial natriuretic peptide, released from cardiac atria in response to mechanical stretch, has been proposed as an important mediator of glycocalyx shedding [72–74]. Alternatively, rapid infusion of intravenous fluid may cause direct endothelial shear stress which may promote the activity of glycocalyx-shedding metalloproteinases [75] or cause neutrophil activation which may result in neutrophil elastase-induced endothelial injury [76, 77].

### Vascular leakage and tissue hypoxia

**Fig. 4:**
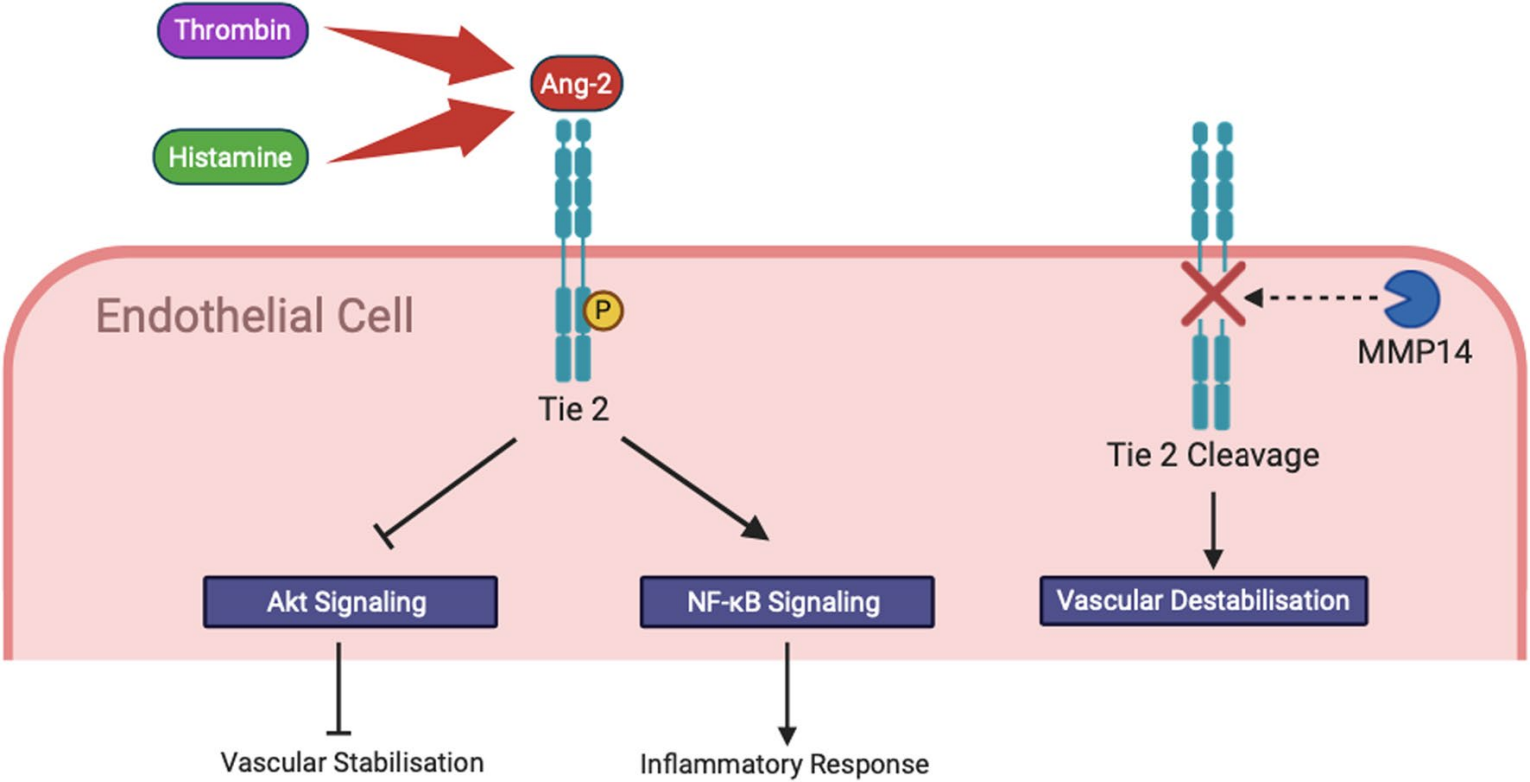
In sepsis Ang-2 acts as an antagonist of Tie 2 which results in disruption of protective Ang-1/Tie 2 signalling. The antagonistic effects of Ang-2 leads to increased inflammation and inhibition of the vascular stabilising Akt signalling pathway. Moreover, the vascular barrier protective effects of Tie 2 are abrogated by the cleaving properties of MMP14

In normal health a functioning and highly selective endothelial barrier is crucial to the maintenance of microvascular homeostasis. The angiopoietin-Tie 2 pathway is a complex, multifaceted cascade which is commonly implicated in vascular permeability. Tie 2 is a transmembrane endothelial tyrosine kinase [78]. Angiopoietin-1 (Ang-1) acts as a Tie 2 agonist and exerts a protective effect on the endothelium by promoting endothelial barrier function [79]. Ang-1, via Akt activation, inhibits the activity of the forkhead transcription factor which is a key regulator of genes associated with endothelial destabilisation [80]. In contrast, Angiopoietin-2 (Ang-2) is a context-dependent Tie 2 agonist or antagonist. The release of Ang-2 from Weibel-Palade bodies can be stimulated by key pro-permeability mediators such as thrombin and histamine [81]. In a murine LPS-induced endotoxaemic model of sepsis, Ang-2 binding resulted in Tie 2 antagonism [82], thus negating the protective effects of Ang-1 (Fig. 4). Moreover, Ang-2 binding to Tie 2 precipitates integrin degradation and endothelial barrier destabilisation [83]. In addition to Tie-2 antagonism, Ang-2 has been revealed to directly activate *β*1-integrin which resulted in cytoskeleton reorganisation and destabilization of intercellular junctions via increased cell contractility [84]. Thamm and colleagues have demonstrated increased Tie-2 cleavage in endothelial cells exposed to TNF- *α*, septic mice and septic humans [85]. Moreover, it was demonstrated that the matrix metalloprotease, MMP14, performed a central role in the cleavage of Tie-2 [85]. Furthermore, in a cecal ligation and puncture (CLP) model the investigators also demonstrated that Tie 2 transcription was dependent on flow [85, 86]. Absent flow, such as that observed in the septic microcirculation, was associated with reduced levels of GATA3, a flow dependent transcription factor which performs a key role in regulating Tie 2 transcription [85, 86].

Importantly, Ang-2 has been identified as a prognostic biomarker in sepsis [87, 88], with Ang 2 levels correlating with disease severity and survival [89].The prominent role of the angiopoietin-Tie 2 pathway in endothelial dysfunction makes modulation of the Ang-1/Ang-2/Tie-2 equilibrium an attractive therapeutic target in sepsis. In a CLP model of sepsis, the use of a synthetic Tie 2 agonist was associated with an attenuated cytokine response, reduced vascular leakage, and improved organ function [90]. In another CLP model the use of Ang-2 small interfering RNA was associated with reduced IL-6 transcription and reduced levels of neutrophil infiltration, vascular leakage, and organ dysfunction [91].

**Fig. 5:**
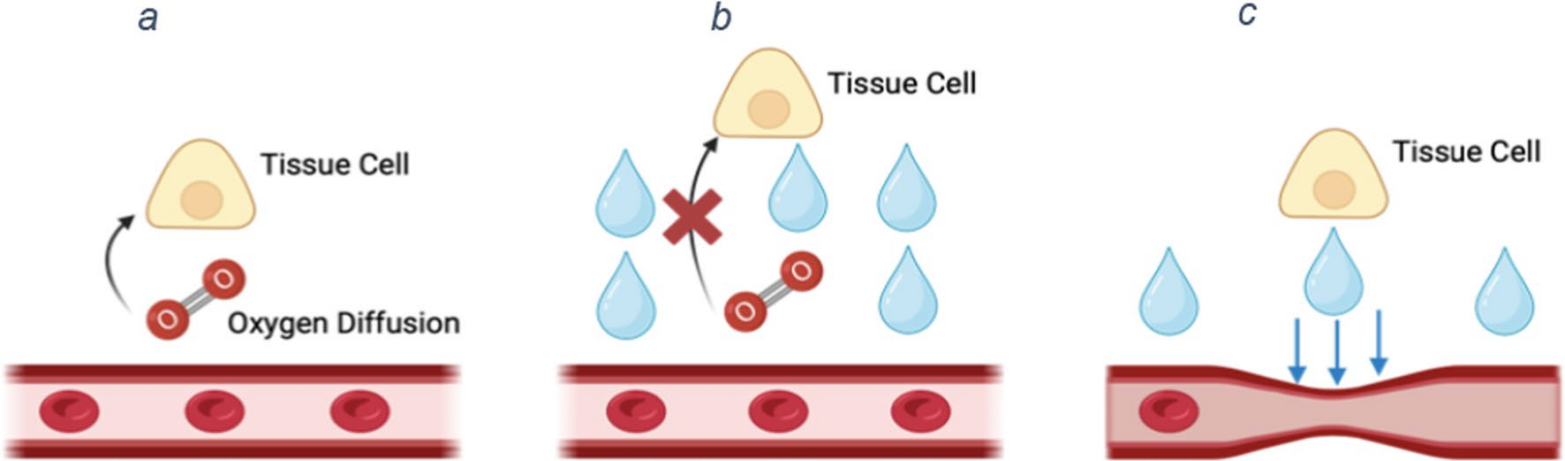
Normal oxygen diffusion from blood vessels to target tissue cells. **b** Tissue hypoxia occurring due to increased diffusion distance between oxygen carrying red blood cells in the microvascular blood vessels and the mitochondria of tissue cells. **c** Tissue hypoxia occurring due to a tamponade like effect of interstitial fluid on microvascular blood vessels

Oxygen delivery occurs via diffusion of oxygen from capillary red blood cells to the mitochondria of tissue cells. Diffusion is dependent on the PO_2_ diffusion gradient between capillaries and tissue cells and on the diffusion distance from capillary red blood cells to tissue cell mitochondria [92]. In sepsis, heterogenous generation of NO, secondary to endothelial dysfunction, results in pathological vasodilatation and shunt formation with ensuant variable perfusion of tissue regions and cellular hypoxia in areas distant from perfused capillaries [92, 93]. Injury to the endothelial glycocalyx and to inter-endothelial junctional structures, culminating in interstitial oedema, may compound this problem as it increases diffusion distance between capillaries and cells (Fig. 5) [92]. Mechanical extrinsic compression of capillaries and lymphatics by interstitial fluid may further worsen oxygen delivery (Fig. 5) [94]. Exacerbation of tissue hypoxia by oedema may explain adverse outcomes associated with fluid overload in patients with sepsis [95–97].

### Potential therapeutic approaches

Given the prominent role of endothelial breakdown and dysfunction in sepsis, preservation and restoration of endothelial function represents a key therapeutic target.

### Imatinib and other tyrosine kinase inhibitors

The Abelson (Abl) family of tyrosine kinases, Abl (Abl1) and Arg (Abl2), perform an important role in cytoskeletal remodelling, adhesion, and migration [98]. Zandy et al., demonstrated the importance of Abl kinases in the formation and maintenance of adherens junctions [99]. The inhibition of tyrosine kinase Arg, also known as Abl2, serves to maintain endothelial barrier integrity. It has been demonstrated that depletion of Arg in endothelial cells is associated with reduced adherens junctions disruption and intercellular gap formation [100].

Imatinib, the most widely-studied Tyrosine Kinase Inhibitor, potentiates the activity of Rac 1 [101, 102], an endothelial barrier-supporting GTPase known to reinforce cell–matrix [103] and cell–cell interactions [104]. Imatinib targets the Abl family of non-receptor tyrosine kinases in addition to other tyrosine kinases, such as platelet-derived growth factor receptor, and the receptor tyrosine kinase Kit [105].

Vascular barrier protective effects of Imatinib have been identified in in vivo models of microcirculatory dysfunction and in patients with endothelial barrier disruption [106, 107]. In addition, the in vivo protective effects of Imatinib may be attributable to the effect on immune cells with Imatinib attenuating inflammation in animal models of LPS-induced lung injury [108, 109]. A potential clinical benefit has been demonstrated in patients with COVID-19, which shares many mechanistic features with sepsis [110]. There is, therefore, a growing body of evidence to support a potential role for the short-term administration of Imatinib as a therapeutic agent to maintain endothelial barrier integrity and attenuate inflammation in sepsis.

### Selepressin

Vasopressin deficiency contributes to vascular dysfunction in septic shock [111] which provides the rationale for investigation of vasopressin receptor agonists in patients with sepsis. To date, however, vasopressin has failed to demonstrate clinical benefit over noradrenaline in sepsis [112]. One possible explanation is that non-specific vasopressin receptor stimulation can result in detrimental microcirculatory effects. Stimulation of endothelial V2 receptors can result in vasodilation via endothelial NOS activation [113], leucocyte adhesion and migration [114], secretion of procoagulant mediators [115] and salt and water retention [116]. 

Selepressin is a selective vasopressin V1a receptor agonist. In an ovine model of sepsis, animals receiving selepressin therapy had reduced vascular leakage compared to non-specific vasopressin receptor agonists and controls [117]. Moreover, selepressin therapy was associated with reduced myocardial and pulmonary tissue concentrations of VEGF and Ang-2 [117]. VEGF, a potent stimulator of vascular leakage, has been shown to increase the expression of Ang-2 in endothelial cells [118]. In sepsis, Ang-2 disrupts protective Tie2 signalling and contributes to endothelial barrier destabilisation [83]. However, despite the promising pre-clinical evidence base, in an RCT of 868 adult patients with septic shock receiving noradrenaline therapy, the use of selepressin did not improve clinical outcomes [119].

### Mesenchymal stromal cells

Mesenchymal stromal cells (MSCs) are pluripotent stem cells that can differentiate into multiple cell types of mesenchymal lineage [120]. MSC treatment is associated with reduced organ dysfunction and coagulopathy in septic mice [121, 122]. Moreover, MSCs protect against LPS and VEGF induced barrier permeability in human umbilical vein endothelial cells (HUVECs) [122, 123]. Mechanistically, MSC treatment results in increased VE-cadherin levels and promotes VE-cadherin / beta-catenin interaction on endothelial cells [123]. The in vivo endothelial barrier protective effects of MSCs have been confirmed in a murine model of haemorrhagic shock where MSC administration resulted in reduced lung oedema and preservation of vascular tight junctions and adherens junctions [124].

A single centre pilot RCT which included 15 neutropenic patients with septic shock demonstrated more rapid haemodynamic stabilisation with prompt vasopressor weaning and improved PaO_2_/FiO_2_ ratios in those treated with MSC therapy [125]. Alp and colleagues subsequently confirmed the safety of MSCs in patients with sepsis and septic shock and identified reduced Sequential Organ Failure Assessment (SOFA) scores in patients receiving MSCs [126]. However, in a phase 1 dose escalation study in nine patients with septic shock, there was no efficacy signal in the MSC treatment arm [127].

The inflammatory-mediated barrier breakdown in ARDS overlaps with sepsis. Administration of mesenchymal stromal cell-derived extracellular vesicles (MSC-EVs) improves barrier integrity of human primary lung epithelial and endothelial cells following exposure to the plasma of patients with a hypoinflammatory ARDS phenotype [128]. Despite conflicting data on the effect of MSC therapy in phase 2 studies [129–134], there is evidence that MSCs have a protective effect on endothelium, providing a supportive rationale for further investigation in sepsis [134].

### Statins

Statins possess an array of important pleiotropic effects [135, 136]. Zheng and colleagues identified that treatment of HUVECs with Simvastatin attenuated LPS-induced endothelial permeability by potentiating the activity of IQ‐GTPase‐activating protein 1, a regulator of cytoskeletal function [137]. Furthermore, in a rat model of endotoxaemia, Simvastatin treatment attenuated hepatic endothelial dysfunction and preserved the antithrombotic properties of sinusoidal endothelial cells disrupted by LPS [138, 139]. Statin therapy has also been shown to modify the activity of endothelial nitric oxide synthase, a key producer of NO, by preventing hypoxia and TNF- *α* induced downregulation [140]. In addition, it has been demonstrated that statin treatment prevents the nuclear translocation of NF-κB in endothelial cells subjected to pro-inflammatory stimuli [141].

A retrospective cohort analysis of hospitalised patients with bacteraemia identified a significant survival benefit in patients with pre-existing statin therapy [142] although this was not confirmed in a subsequent RCT by Kruger et al. [143]. However, continued statin therapy in patients with pre-existing use is associated with improved survival [143].

### PCSK-9 inhibitors

Proprotein Convertase Subtilisin/Kexin-9 (PCSK-9) inhibitors are an emerging drug class with a growing evidence base for prevention of cardiovascular events in hypercholesterolaemia. PCSK-9 inhibitors inhibit the serine protease PCSK-9 and interfere with the LDL receptor recycling pathway, ultimately leading to recycling of the receptor and increased LDL cholesterol clearance [144]. PCSK-9 levels are elevated in patients with sepsis [145] and expression is upregulated in various models of sepsis [146]. Moreover, PCSK-9 knockout is associated with reduced bacterial dissemination, organ dysfunction and inflammation in a murine model of sepsis [147]. Importantly, PCSK-9 inhibition reverses impaired VE-cadherin expression observed in in vitro and in vivo models of sepsis [146].

Similar to statins, PCSK-9 inhibitors possess pluripotent properties. PSCK-9 deficient mice exhibit reduced expression of NADPH oxidase, a major source of ROS production which may further serve to protect the endothelium [148]. The anti-inflammatory effects of PCSK-9 inhibition have been demonstrated by Tang and colleagues who confirmed that in vitro PCSK-9 inhibition attenuates the generation of inflammatory cytokines by interfering with the NF-kB pathway [149].

In a placebo controlled, multicentre pilot trial, 60 patients with severe COVID-19 infection were randomised to receive 140-mg subcutaneous injection of Evolocumab, a PCSK-9 inhibitor, or placebo [150]. The investigators demonstrated that compared to placebo, PCSK-9 inhibition resulted in a greater reduction in IL-6 levels, a reduced requirement for invasive ventilation and improved mortality. This highlights the potential role of PCSK-9 inhibitors as endothelial barrier protective agents.

### Alpha adrenoceptor agonists

Alpha adrenoceptor agonists such as Clonidine and Dexmedetomidine cause sympathetic inhibition and parasympathetic stimulation [151]. The expression of adrenergic receptors on endothelial cells provides a sound rationale for investigation of these agents.

Dexmedetomidine is a commonly used sedative which attenuates inflammatory cytokine production in septic patients [152]. In a LPS rat model of endotoxaemia, Dexmedetomidine administration was associated with attenuated TNF- α and IL-6 levels and a reduction in mortality [153]. This anti-inflammatory and mortality benefit has also been observed in CLP models of sepsis [154]. Moreover, Yeh and colleagues have demonstrated that Dexmedetomidine reduces tight junction damage, endothelial dysfunction, and microcirculatory impairment in endotoxaemic rats [155].

Similarly, there is a growing body of evidence to support the investigation of Clonidine in sepsis. In a CLP murine model of sepsis, the pre-emptive administration of Clonidine attenuated pro-inflammatory cytokine release, downregulated the binding activity of NF-κB, and reduced mortality [156]. Moreover, Schmidt and colleagues confirmed that Clonidine administration was effective in attenuating microvascular permeability in endotoxaemic rats [157].

### Intermedin

Intermedin is a member of the calcitonin gene related peptide family which exerts its effects via the calcitonin receptor-like receptor signalling pathway [158]. Aslam and colleagues identified that Intermedin reduces HUVEC permeability and induces Rac1 activation, a key endothelial barrier supporting GTPase [159]. It has been determined that pre-treatment of mice with Intermedin attenuates vascular leakage in LPS and CLP models of sepsis [160]. Furthermore, the anti-inflammatory effects of Intermedin have been demonstrated in a CLP model of sepsis in which Intermedin tempered inflammatory cytokine production [160].

### Adrenomedullin

Adrenomedullin (ADM), another member of the calcitonin gene related peptide family, is a vasoactive peptide hormone which regulates endothelial barrier function and vascular tone. It has been demonstrated that blood ADM levels correlate with vasopressor requirement and mortality in patients with sepsis [161, 162].

In vitro data has confirmed that ADM attenuates endothelial permeability in HUVECs [163, 164]. Moreover, in a Staphylococcus aureus toxin model of sepsis in rats, administration of ADM attenuated endothelial leakage and reduced mortality from 53 to 7% [165]. These outcomes suggest that ADM performs a key role in controlling endothelial barrier function and vascular tone, however, meticulous regulation is required. Of note, a multicentre phase 2 RCT investigating the safety and efficacy of inhaled pegylated adrenomedullin in adult patients with ARDS was recently stopped prematurely due to futility (NCT 04417036).

### Adrecizumab

Adrecizumab is a non-neutralising ADM binding antibody which targets the N-terminus of ADM and only partially inhibits ADM signalling. In a CLP murine model of sepsis, Struck and colleagues demonstrated that the partial inhibition of ADM was more efficacious than an antibody which completely blocked ADM [166]. The investigators hypothesised that partial functional inhibition of ADM negates the harmful effects of excessive ADM while still preserving an adequate degree of ADM activity which may be required, especially in the early hyperdynamic phase of sepsis [166].

The endothelial barrier protective effects of Adrecizumab have been demonstrated in LPS and CLP rat models of inflammation and sepsis [167]. The AdrenOSS-2 trial, a biomarker-guided randomised trial, compared Adrecizumab with placebo in patients with septic shock and elevated concentrations of ADM. AdrenOSS-2 revealed that Adrecizumab was associated with a greater improvement in SOFA scores compared to placebo and demonstrated a trend towards decreased mortality (23.9% versus 27.7%) [168]. Although promising, further trials of Adrecizumab are needed.

### Vitamin C

Vitamin C has been extensively investigated in the management of sepsis. Zhou et al., demonstrated that Vitamin C pre-treatment in a CLP model of sepsis reduced excessive production of NO and ROS and attenuated vascular leakage by preventing the dephosphorylation of occludin [169]. Occludin dephosphorylation results in disassembly of tight junctions and increased vascular permeability [170]. Pre-clinical and clinical evidence has highlighted that Vitamin C attenuates endotoxin induced lung injury [171] and oedema formation in patients with burn injuries [172]. However, despite a promising pre-clinical evidence base, results from RCTs investigating Vitamin C have been disappointing. A recent meta-analysis which included 37 trials concluded that parenteral Vitamin C therapy was not associated with a mortality benefit [173].

### Canaglifozin

Canaglifozin is a sodium glucose co-transporter 2 inhibitor utilised in the management of diabetes mellitus. Canaglifozin is an activator of AMPK, a serine/threonine protein kinase, which exerts a protective effect on endothelial adherens junctions and tight junctions [174, 175]. Moreover, it has been established that Canaglifozin attenuates LPS induced vascular leakage in mice [176]. No clinical trials of Canaglifozin have been undertaken in sepsis to date.

### Humanin

Mitochondrial derived peptides, such as Humanin, possess key biological properties which make them an attractive therapeutic strategy in sepsis. Humanin has potent cytoprotective properties and has been demonstrated to protect endothelial cells from hyperglycaemia and oxidative stress [177, 178]. Humanin may mediate this protection via increased expression of Krüppel-like factor 2, an important transcriptional regulator of endothelial function [177].

Urban and colleagues have recently determined that a synthetic derivative of humanin, Colivelin, protects against endothelial injury and glycocalyx damage in a murine CLP model of sepsis [179]. It was highlighted that Colivelin activates AMPK which may be responsible for the vascular protective effects observed in the treatment group.

### Fresh frozen plasma (FFP)

Fresh Frozen Plasma (FFP) is used to correct clotting factor deficiencies in bleeding patients and in coagulopathic patients at risk of bleeding [180]. Some studies have identified reduced mortality following FFP administration irrespective of correction of the underlying coagulopathy [181, 182]. Therefore, in addition to correcting coagulation factor deficiencies it has been postulated that FFP may possess endothelial protective properties. Straat and colleagues investigated the effects of FFP administration in non-bleeding critically ill patients, half of whom had sepsis [183]. FFP treatment was associated with reduced syndecan-1 and factor VIII levels, potentially reflecting attenuation of endothelial injury [183].

### Activated protein C

Activated protein C (APC) is an endogenous protein generated from an inactive precursor, protein C, via the action of the Thrombin-Thrombomodulin complex [184]. APC possesses anti-coagulant and anti-inflammatory actions and has been demonstrated to inhibit neutrophil chemotaxis [185] and prevent endothelial cell apoptosis [186]. These properties made APC an attractive therapeutic option to investigate in sepsis.

Feistritzer and Riewald identified that the thrombin induced hyperpermeability of HUVECs was attenuated with APC pre-treatment [187]. However, the in vivo effects of APC on endothelial permeability are conflicting [188, 189].

The first phase 3 study to investigate APC in sepsis included 1690 patients with severe sepsis [190], and reported significantly reduced mortality at 28-days (30.8% in the placebo group vs 24.7% in the APC group) [190], following which APC received marketing authorisation and approvals in patients with severe sepsis who were considered at high risk of mortality. However, subsequent trials failed to confirm these results [191, 192], and worldwide withdrawal of APC from the market followed.

In a recent secondary analysis of the PROWESS-SHOCK trial, Sinha and colleagues tested for heterogeneity of treatment effect in inflammatory phenotypes [193]. The investigators revealed that APC treatment was associated with a higher 28-day mortality in the hypoinflammatory phenotype (APC 24.3% vs Placebo 19.5%), whereas mortality was reduced in the hyperinflammatory phenotype (APC 33.0% vs Placebo 41.3%) [193]. APC may have been a victim of the phenotypic heterogeneity which besets sepsis.

## Conclusion

Microvascular dysfunction is strongly associated with morbidity and mortality in sepsis. Microcirculatory dysfunction encompasses distinct pathological processes such as abnormal NO expression with ensuant heterogenous capillary perfusion, increased endothelial adhesiveness to leucocytes and platelets, dysregulation of smooth muscle cells with a loss of adrenergic sensitivity and increased endothelial permeability. This review has focused on the endothelial permeability aspect of microcirculatory dysfunction.

Despite the detrimental effects of vascular dysfunction and endothelial breakdown, no pharmacological therapies are currently used to attenuate vascular leakage. When putative endothelial protective agents have been studied in RCTs to date the results have been disappointing despite promising pre-clinical evidence. Future investigation of these agents should involve targeted treatment of endothelial injury in mechanistically orientated trials with a homogenous patient population. Ideally, this would involve development of diagnostic methods to facilitate rapid diagnosis and phenotyping of endothelial dysfunction and would provide a platform for observation of response to treatment alongside patient-centred clinical outcomes.

### Acknowldgements

DM reports grants from NIHR, Innovate UK, MRC, Northern Ireland HSC R&D division, Wellcome Trust, Randox and Novavax as an investigator in ARDS and COVID-19 studies. DM reports consultancy fees unrelated to this work from Bayer, GlaxoSmithKline, Aptarion, Direct Biologics, Aviceda Boehringer Ingelheim, Novartis SOBU, and Eli Lilly. DM reports payments from GlaxoSmithKline as an educational seminar speaker. DM reports fees as a member of the DSMB for Vir Biotechnology, Inc and Faron Pharmaceuticals. DM has a patent for a novel treatment for inflammatory disease. DM was a Director of Research for the Intensive Care Society, Director of the MRC/NIHR EME programme and NIHR Scientific Director for Programmes. DM reports his spouse has received consultancy fees from INSMED and from the California Institute for Regenerative unrelated to this work. CO reports grants from the Wellcome Trust, MRC, NI HSC R&D Division, Innovate UK as an investigator in ARDS and COVID studies. CO reports consultancy from INSMED unrelated to this work and fees for grant panel membership from the Californian Institute of Regenerative Medicine. CO spouse reports (a) consultancy fees unrelated to this work from Bayer, GlaxoSmithKline, Aptarion, Direct Biologics, Aviceda Boehringer Ingelheim, Novartis SOBU, and Eli Lilly (b) payments from GlaxoSmithKline as an educational seminar speaker (c) fee as a member of the DSMB for Vir Biotechnology, Inc and Faron Pharmaceuticals(d) a patent for a novel treatment for inflammatory disease. CO spouse was a Director of Research for the Intensive Care Society, Director of the MRC/NIHR EME programme and NIHR Scientific Director for Programmes. JS reports grants from MRC, NI HSC R&D Division, NIAA and NIHR related to studies in sepsis. JS has received consultancy fees or honoraria unrelated to this work from Edwards Lifesciences, Baxter Healthcare, and Merck Sharpe Dolme, and research support from CASMED (now Edwards Lifesciences).

## Data Availability

Not Applicable.

## Abbreviations

Abl: Abelson
ADM: Adrenomedullin
Ang-1: Angiopoietin-1
Ang-2: Angiopoietin-2
APC: Activated protein C
ARDS: Acute respiratory distress syndrome
CLP: Cecal ligation and puncture
HUVECs: Human umbilical vein endothelial cells
IL: Interleukin
LPS: Lipopolysaccharide
MSCs: Mesenchymal stromal cells
MSC-EVs: Mesenchymal stromal cell-derived extracellular vesicles
NF-κB: Nuclear factor-κB
NO: Nitric oxide
PCSK-9: Proprotein Convertase Subtilisin/Kexin-9
RCT: Randomised control trial
ROS: Reactive oxygen species
SOFA: Sequential Organ Failure Assessment
TNF-α: Tumour necrosis factor alpha
VE-cadherin: Vascular-endothelial cadherin
VEGF: Vascular endothelial growth factor
ZO: Zonula occludens
